# Hygienic Conditions at the Crystal Palace: Have Sufficient Precautions Been Taken?

**Published:** 1915-02-27

**Authors:** 


### HYGIENIC CONDITIONS AT THE CRYSTAL PALACE.

### Have Sufficient Precautions been Taken?

(FROM A CORRESPONDENT.)

The timely question asked in the House of Commons, by Mr. Joynson Hicks, the member for Brentford, elicited from the Secretary to the Admiralty a statement concerning the health of the large body of men lodged in the Palace and its grounds, which, in face of the persistent and alarming rumours circulating in the district, is to a great extent reassuring, although some cause for anxiety remains. The abrupt closing of the Palace to the public, including season-ticket holders, and the absence of all explanation, led, as might have been expected, to sinister reports. The official announcement that of 6,578 officers and men in residence not more than 184 were upon the sick list goes far to correct these, and would be quite satisfactory but for the fact that fifteen cases of spotted fever (cerebro-spinal meningitis) had occurred, of which eight proved fatal.

If, regarded superficially, the number of such cases in relation to the whole body of men is not large, the pertinent question is why are there any? The Admiralty disclaim knowledge of any cases having occurred in the neighbourhood of the Palace, and no one has suggested that the disease originated among the civilian population. Therefore we must seek among the occupiers of the Palace, and possibly in the hygienic conditions surrounding them, for elucidation of the problem.

As The Hospital readers know, doubtless, the Crystal Palace was taken over by the Admiralty in September last, and since has been occupied by a large and increasing force of men and youths in training for the Royal Navy. The recruits arrive in squads collected from all parts of the country, and apparently proceed at once to mingle with their future comrades. Naturally their personalities show great diversity; some of the recruits are ill-clad, and come, obviously, from poor and possibly unhealthy homes. The question arises whether sufficient precautions are taken to prevent the entry of individuals carrying infection, and the question applies to all forms of infectious disease. The men are lodged largely in the temporary buildings erected three or four years ago for the so-called Festival of Empire. Some of these structures have been already removed after falling into a condition of dilapidation; the larger, and possibly the more substantial, remain. None of them was intended for habitation; they were put up as showrooms where the natural and manufactured products of the several Colonies and Dominions were displayed, and no regard was had to the hygienic requirements of permanent barracks, for which they were never intended. Their walls were constructed mostly of lath and plaster, with roofs of felt or some such material.

Moreover, in addition to these, the Palace itself, little better suited to habitation, is fully utilised, sleeping accommodation being provided in the galleries and elsewhere. The ground floor, cleared of its statuary, goods stalls, and other impedimenta, is used for drill, instruction classes, etc. The concert room supplies a spacious refectory; other chambers for taking meals are provided by screening off wide spaces on the outskirts of the vast nave, while the many fine courts which open from it on the west side have become, at the instance of the Y.M.C.A., reading, writing, and recreation rooms, with post offices, a chapel, officers' quarters, and other needful accessories of a great camp. In the grounds many fields for football and other outdoor games have been provided.

Upon the whole, the building has lent itself readily to its new rôle. To the onlooker the arrangements appear admirable, with evidences in plenty that the well-being of the men has been studied, while in conversation the sailors themselves have been quick to acknowledge the general comfort of their quarters.

But without thorough and expert inspection it would be difficult to say how far the existing needs of sanitation have obtained attention. To provide for them adequately was in itself a great undertaking which could hardly have been overlooked by competent authority, and what has already occurred, apart from the possibilities of development, always present when infectious disease has obtained a footing, calls for some authoritative information of what has been done, and is being done, by way of safeguard. This is urged in no carping spirit and with a full sense of the difficulties the authorities have encountered when called upon to make hasty provision for the housing of vast bodies of troops.

In the circumstances, nobody would object to the use of the Crystal Palace for its present purpose, even though with less urgent need it might not have appeared structurally suitable. But in view of the importance of the subject in all its bearings, the public have a right to ask whether the resources of modern sanitary science have been duly utilised to overcome the inherent defects of buildings applied to uses for which they were not designed.

